# Various ambient air pollutants, residential greenness, and risk of substance use disorders: a population-based cohort study

**DOI:** 10.1186/s12888-026-08588-8

**Published:** 2026-09-09

**Authors:** Huan Liu, Bolun Cheng, Wenming Wei, Lina Qin, Feng Zhang

**Affiliations:** 1 NHC Key Laboratory of Environment and Endemic Diseases, Xi’an Jiaotong University, Xi’an, 710061 China; 2 Collaborative Innovation Center of Endemic Disease and Health Promotion for Silk Road Region, School of Public Health, Health Science Center, Xi’an Jiaotong University, Xi’an, 710061 China; 3 Department of Psychiatry, The First Affiliated Hospital of Xi’an Jiaotong University, Xi’an, 710061 China

**Keywords:** Air pollution, Green environment, Substance use disorders, Prospective cohort

## Abstract

**Background:**

Air pollution is a recognized contributor to the global burden of disease, but its association with incident substance use disorders (SUD) remains poorly characterized. Whether residential greenness modifies this association is also unclear. We addressed both questions in a large prospective cohort.

**Methods:**

We analyzed 247,138 UK Biobank participants without SUD at baseline. Five ambient air pollutants (PM_2.5_, PM_2.5−10_, PM_10_, NO_2_, NO_X_) and three measures of 300-m residential greenness (green space, domestic garden, natural environment) were modelled using Land Use Regression and the 2005–2007 Land Cover Map. Cox proportional hazards models estimated hazard ratios with 95% confidence intervals. Effect modification by greenness was tested using cross-product terms and likelihood-ratio tests. Sensitivity analyses restricted to long-term residents, used alternative outcome definitions (F10-F19 and F11-F19), and recomputed greenness at a 1000-m buffer.

**Results:**

Over a median follow-up of 10.9 years, 4,982 participants developed SUD. All five air pollutants were positively associated with incident SUD in fully adjusted models. PM_2.5_ showed the strongest effect (HR = 1.111, 95% CI: 1.081–1.142), followed by PM_10_ (HR = 1.070, 95% CI: 1.054–1.086). Green space (HR = 0.997, 95% CI: 0.996–0.998) and natural environment (HR = 0.997, 95% CI: 0.995–0.998) were inversely associated. Domestic garden attenuated the pollutant associations in the high-exposure subgroup, with cross-product terms of 0.979 (95% CI: 0.963–0.996) for PM_2.5_ and 0.987 (95% CI: 0.980–0.995) for PM_10_. The positive air-pollution associations were strongest among males, participants aged ≥ 65 years, and individuals with a history of smoking.

**Conclusion:**

Long-term exposure to ambient air pollutants was associated with a higher risk of incident SUD, and residential greenness showed protective associations. These patterns were most pronounced in older males and in individuals with a smoking history. The findings support integrating greenness exposure into environmental frameworks for SUD prevention.

**Clinical trial number:**

Not applicable.

**Supplementary Information:**

The online version contains supplementary material available at https://doi.org/10.1186/s12888-026-08588-8.

## Background

Substance use disorders (SUD) are persistent patterns of alcohol or drug consumption that produce functional or clinical impairment [1]. Globally, approximately 300 million people live with SUD [2], and the condition imposes a substantial burden on public health systems. Twin and family studies suggest moderate heritability, with estimates ranging from 0.04 to 0.12 [3], which leaves most of the variation attributable to environmental exposures. Identifying modifiable environmental drivers has therefore become a pressing public-health priority [4].

Ambient air pollution is one such driver. Particulate matter is a leading contributor to the global burden of disease, accounting for an estimated 2.9 million deaths annually [5], and observational data increasingly link air pollutants to a range of psychiatric outcomes [6, 7]. Studies on air pollution and substance use disorders have so far been predominantly cross-sectional, with inconsistent results and limited sample sizes, and prospective evidence therefore remains sparse.

Residential greenness has emerged as a candidate protective factor. Cross-sectional and longitudinal studies link green-space exposure to lower risks of adverse birth outcomes [8], chronic diseases [9], and premature mortality [10], and recent UK Biobank analyses suggest benefits for incident psychiatric disorders [11]. Mechanistically, green spaces may attenuate exposure to air pollutants, buffer noise and heat stress, support physical activity, and foster social cohesion. Whether greenness modifies the relationship between air pollution and SUD has not been tested prospectively.

In this study, we addressed this gap using UK Biobank data on five ambient air pollutants, three measures of residential greenness, and incident SUD. We tested the main effects of each exposure, the joint effects of pollutants and greenness, and effect modification by sex, age, body mass index, smoking, alcohol intake, and prior illicit-substance use.

## Methods

### Study participants

**Fig. 1:**
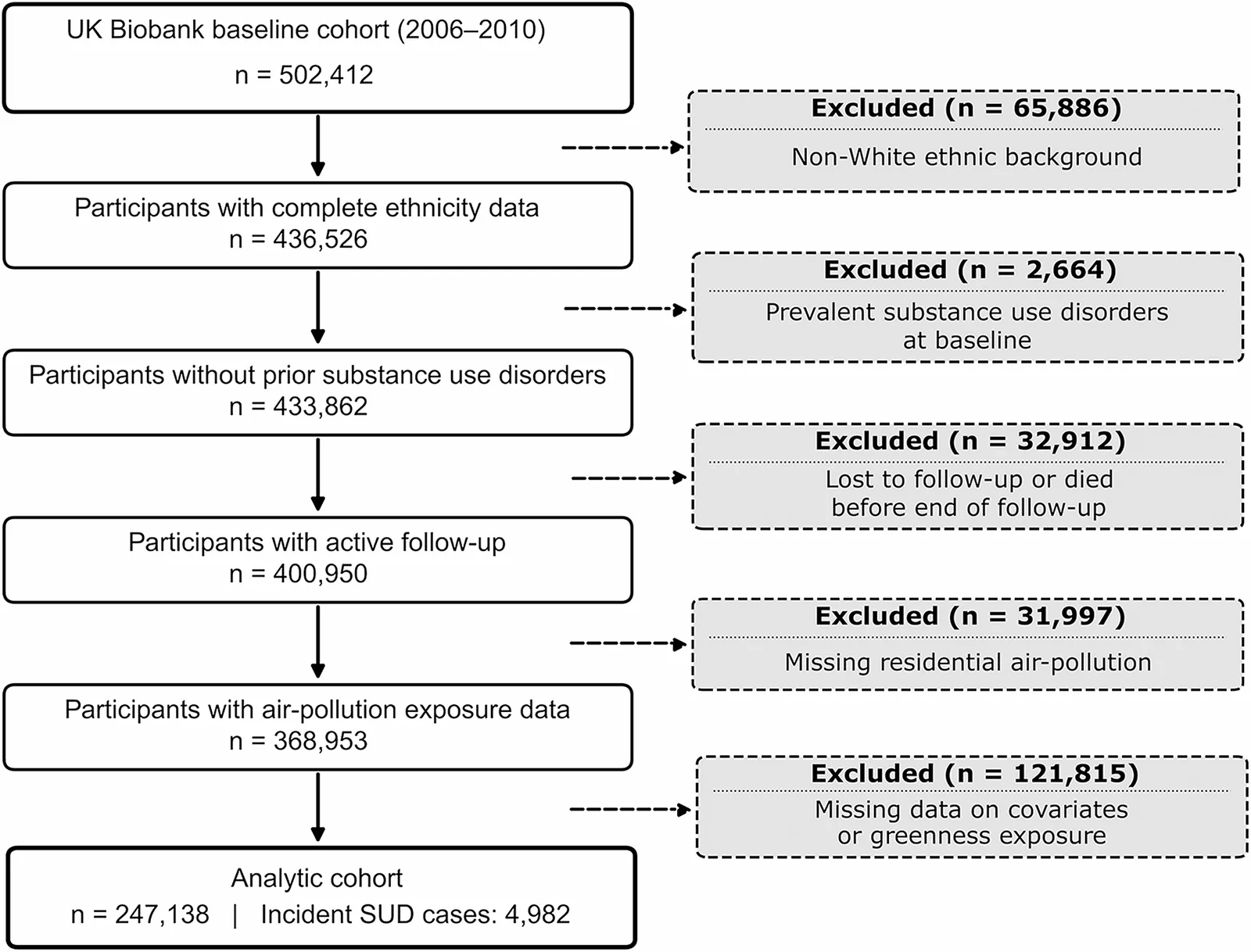
Participant selection in this study. SUD, substance use disorders

This study used data from the UK Biobank, a prospective cohort of more than 500,000 UK residents aged 37–73 years who were enrolled between 2006 and 2010 at 22 assessment centers across England, Wales, and Scotland. The UK Biobank received approval from the North West Multi-Centre Research Ethics Committee, and all participants provided written consent. Further cohort details have been reported elsewhere [12]. From the 502,412 participants assessed at baseline, we excluded individuals with prevalent SUD and those with missing data on any environmental exposure (the five air pollutants and the three greenness measures) or any covariate (age, sex, body mass index, Townsend deprivation index, educational attainment, smoking status, alcohol intake, and duration of residence). The analytic sample comprised 247,138 participants (Fig. 1).

### Assessment of substance use disorders

Incident SUD were ascertained through hospital inpatient records, outpatient treatment, and primary care records linked to Health Episode Statistics (England and Wales) and the Scottish Morbidity Records (Scotland). The earliest recorded diagnosis served as the index event, and follow-up was censored on 30 September 2021. Cases were identified using International Classification of Diseases, Tenth Revision (ICD-10) codes F10, F11, F12, F13, F14, F15, F16, and F19 (Supplementary Table S1). F17 (tobacco use disorder) was excluded because tobacco use is both a potential source of air-pollution exposure and a downstream outcome in this setting, which would introduce reverse-causation confounding [13]. F18 (volatile solvent use disorder) was excluded because volatile solvents are themselves a constituent of ambient air pollution, which would create overlap between exposure and outcome [14]. Within the F10-F16 and F19 code blocks we retained all sub-codes (F1x.0 acute intoxication, F1x.1 harmful use, F1x.2 dependence syndrome, F1x.3 withdrawal state, F1x.4 withdrawal state with delirium, F1x.5 psychotic disorder, F1x.6 amnesic syndrome, F1x.7 residual and late-onset psychotic disorder, F1x.8 other mental and behavioral disorders, and F1x.9 unspecified mental and behavioral disorder), which yielded a comprehensive case definition.

### Estimation of air pollution exposure

Annual average concentrations of PM_2.5_, PM_2.5−10_, PM_10_, and NO_X_ in 2010, and NO_2_ in 2005–2007 and 2010, were modelled for each participant’s residential address using Land Use Regression (LUR) model developed as part of the European Study of Cohorts for Air Pollution Effects (ESCAPE) [15, 16]. The LUR models drew on Geographic Information System (GIS) predictor variables such as traffic intensity, land use, and topography, and produced annual average concentrations of air pollutants [15]. The ESCAPE project developed LUR models that calculated annual moving average concentrations of air pollutants based on GIS variables at a 100 m × 100 m spatial resolution [17]. Full details of the model development and validation procedures are reported in the original ESCAPE publications [15, 16].

### Estimation of green environments

Residential greenness was assessed as the percentage coverage of green space, domestic garden, and natural environment within a 300-m circular buffer around each participant’s home at baseline [18]. The 300-m buffer was chosen a priori on the basis of established accessibility standards and neurobiological plausibility. The WHO recommends 300 m, roughly a 5-min walk, as the maximum acceptable distance to green space, a threshold embedded in the widely adopted 3-30-300 rule [19]. At the neurobiological level, brief nature exposure (≤ 15 min) acutely restores prefrontal cortex function and reduces cortisol and sympathetic activity [20]. Residential green-space and domestic-garden coverages were derived from the 2005 Generalized Land Use Database at the 2001 Census Output Area level. Natural-environment coverage was derived from the 2007 Land Cover Map produced by the Centre for Ecology and Hydrology [21]. Green space and natural environment partly overlapped by definition. Each participant’s home-location polygon was used to compute an area-weighted mean of land-use percentage coverage.

### Covariates measurements

Covariates were selected on the basis of prior mental-disorder literature and included age, sex, body mass index (BMI), Townsend deprivation index (TDI), educational attainment, smoking status, alcohol intake, and duration of residence. Date of birth and sex were obtained from local NHS Primary Care Trust registries. Height and weight were measured by trained nurses to derive BMI. TDI was computed from residential postcodes as an area-level indicator of socioeconomic deprivation, with higher scores indicating greater deprivation and lower socioeconomic status [22]. Educational attainment was classified on a five-level scale based on the UK Biobank dataset (data field 6138), ranging from low to high. Smoking, alcohol intake, and duration of residence were collected through touchscreen questionnaires at baseline.

### Statistical analysis

Cox proportional hazards regression was used to estimate hazard ratios (HR) with 95% confidence intervals (CI) for the associations of air pollutants and greenness with incident SUD. Two models were fitted. Model 1 adjusted for age and sex. Model 2 (the primary model) additionally adjusted for BMI, TDI, educational attainment, smoking status, alcohol intake, and duration of residence. E-values were computed for Model 2 as a quantitative sensitivity measure for unmeasured confounding [23]. Four pre-specified sensitivity analyses were conducted. First, we restricted the sample to long-term residents (duration of residence > 10 years, UK Biobank field 699) to minimize exposure misclassification due to recent residential mobility. Second, we used all ICD-10 sub-codes within F10-F19 for the composite outcome. Third, we restricted the outcome to illicit substance use disorders (F11-F19) to address potential over-adjustment for alcohol-related behavior. Fourth, we recomputed all greenness metrics using a 1000-m buffer.

Dose-response relationships between air pollutants, green environments, and SUDs risk were examined using restricted cubic spline (RCS) regressions [24]. To balance model fitting and overfitting, a range of 3–5 knots was initially considered for constructing the main splines. The optimal number of knots was determined using Akaike’s Information Criterion (AIC) [25].

Effect modification by green environment was tested by categorizing greenness into low (1st quartile), moderate (2nd -3rd quartiles), and high (4th quartile) groups, and adding cross-product terms between each air pollutant (continuous) and the greenness category to the Cox models. The statistical significance of multiplicative interaction was assessed using likelihood-ratio tests comparing models with and without the cross-product terms.

Stratified analyses were conducted by age (< 65 vs. ≥ 65 years), sex (female vs. male), BMI (normal: 18.5–25 kg/m^2^; overweight: 25.0–30.0 kg/m^2^; obese: ≥30.0 kg/m^2^), smoking status (never vs. ever), alcohol drinking (never vs. ever), and illicit-substance use (yes vs. no). Formal multiplicative interaction within each stratum was tested using likelihood-ratio tests comparing models with and without cross-product terms between the stratification variable and the exposure of interest. All analyses used R software (version 4.1.0), with a two-sided *P* value threshold of 0.05. Cox models were fitted with the survival package, RCS analyses with the rms package, and figures with the forestplot and ggplot2 packages.

## Results

### Characteristics of study participants

Table 1 presents the baseline characteristics of the analytic sample. Among 247,138 participants, 4,982 developed an incident substance use disorder over follow-up. The cumulative person-years at risk were 54,253 for cases and 2,617,476 for non-cases. Compared with non-cases, participants who developed a substance use disorder were more often female (68.25% vs. 46.27%), more often had a history of smoking (61.54% vs. 33.73%) or any alcohol intake (99.20% vs. 95.64%), and had a marginally higher mean BMI (27.81 vs. 27.14 kg/m^2^). Participants who developed a substance use disorder also had higher exposure to all five air pollutants and lower exposure to all three greenness measures.

**Table 1:** Baseline characteristics of participants in the UKB study of the association of air pollution and green environment with substance use disorder

Variables	Substance use disorder	
	Incident (*n* = 4,982)	Non-incident (*n* = 242,156)
Total person years at risk, years	54,253	2,617,476
Median follow-up time, years	10.86	10.78
Age, years (mean ± SD)	55.91 ± 8.12	56.29 ± 7.95
Sex, *n* (%)		
Female	3,400 (68.25)	112,045 (46.27)
Male	1,582 (31.75)	130,111 (53.73)
Body mass index [kg/m^2^ (mean ± SD)]	27.81 ± 4.85	27.14 ± 4.50
Smoking, *n* (%)		
Never	1,916 (38.46)	160,487 (66.27)
Ever	3,066 (61.54)	81,669 (33.73)
Alcohol intake, *n* (%)		
Never	40 (0.80)	10,551 (4.36)
Ever	4,942 (99.20)	231,605 (95.64)
Townsend deprivation index, (mean ± SD)	-0.43 ± 3.41	-1.76 ± 2.76
Duration of residence, (mean ± SD)	15.71 ± 12.49	17.43 ± 12.23
Air pollution, µg/m^3^ (mean ± SD)		
PM_2.5_	10.22 ± 1.11	9.90 ± 1.02
PM_2.5−10_	6.48 ± 0.94	6.40 ± 0.90
PM_10_	19.61 ± 1.93	19.09 ± 1.87
NO_2_	30.81 ± 9.25	28.18 ± 8.58
NO_X_	18.67 ± 9.67	16.73 ± 8.51
Green environment, 300 m buffer % (mean ± SD)		
Green space	32.89 ± 21.74	36.85 ± 23.76
Domestic garden	30.09 ± 14.24	31.37 ± 14.93
Natural environment	23.53 ± 23.73	28.29 ± 25.86

The person-years at risk refers to the cumulative follow-up time contributed by each participant, calculated from baseline to the date of SUD diagnosis (for cases), or the earliest terminated time of the study due to loss to follow-up, death, or the end of the study period (September 30, 2021). Abbreviations: UKB, UK Biobank; PM_2.5_, particulate matter with an aerodynamic diameter ≤ 2.5 μm; PM_2.5−10_, particulate matter with an aerodynamic diameter between 2.5 μm and 10 μm; PM_10_, particulate matter with an aerodynamic diameter of 10 μm; NO_2_: nitrogen dioxide; NO_X_, nitrogen oxides

### Longitudinal association of air pollution and green environment exposure with substance use disorders

Table 2 shows the associations of each air pollutant and each greenness measure with incident SUD. In Model 1 (age- and sex-adjusted), all five air pollutants were positively associated with risk. These associations remained statistically significant in Model 2, with HRs of 1.111 (95% CI 1.081–1.142) for PM_2.5_, 1.049 (95% CI 1.019–1.081) for PM_2.5−10_, 1.070 (95% CI 1.054–1.086) for PM_10_,1.013 (95% CI 1.010–1.016) for NO2, and 1.006 (95% CI 1.003–1.009) for NO_X_. For greenness, green space and natural environment were inversely associated with risk in Model 1. The inverse associations persisted in Model 2 for green space (HR = 0.997, 95% CI 0.996–0.998) and natural environment (HR = 0.997, 95% CI 0.995–0.998). Domestic garden showed no statistically significant association in Model 2.

**Table 2:** Association between long-term exposure to air pollution and green environment with substance use disorders

Exposure		Model 1		Model 2	
		HR (95% CI)	*P*-value	HR (95% CI)	*P*-value
Air pollution	PM_2.5_	1.294 (1.264–1.325)	**7.18 × 10** ^**− 102**^	1.111 (1.081–1.142)	**3.24 × 10** ^**− 14**^
	PM_2.5−10_	1.097 (1.066–1.128)	**2.13 × 10** ^**− 10**^	1.049 (1.019–1.081)	**1.55 × 10** ^**− 3**^
	PM_10_	1.152 (1.136–1.168)	**1.21 × 10** ^**− 85**^	1.070 (1.054–1.086)	**2.44 × 10** ^**− 18**^
	NO_2_	1.031 (1.029–1.034)	**8.16 × 10** ^**− 105**^	1.013 (1.01–1.016)	**1.59 × 10** ^**− 14**^
	NO_X_	1.019 (1.016–1.021)	**9.64 × 10** ^**− 54**^	1.006 (1.003–1.009)	**3.30 × 10** ^**− 5**^
Green environment	Green space	0.992 (0.991–0.994)	**1.26 × 10** ^**− 31**^	0.997 (0.996–0.998)	**3.37 × 10** ^**− 6**^
	Domestic garden	0.995 (0.993–0.997)	**2.06 × 10** ^**− 7**^	0.999 (0.997-1.000)	5.88 × 10^− 1^
	Natural environment	0.992 (0.991–0.993)	**2.90 × 10** ^**− 38**^	0.997 (0.995–0.998)	**1.34 × 10** ^**− 7**^

Model 1 was adjusted for age and sex. Model 2 was adjusted for age, sex, BMI, TDI, educational attainment, smoking status, alcohol consumption, and duration of residence. Abbreviations: UKB, UK Biobank; PM_2.5_, particulate matter with an aerodynamic diameter ≤ 2.5 μm; PM_2.5−10_, particulate matter with an aerodynamic diameter between 2.5 μm and 10 μm; PM_10_, particulate matter with an aerodynamic diameter of 10 μm; NO_2_: nitrogen dioxide; NO_X_, nitrogen oxides

E-values indicated that an unmeasured confounder would need to be associated with both exposure and outcome by a risk ratio of at least 1.46 (lower-CI E-value = 1.38) to fully explain the PM_2.5_ association (HR 1.111, 95% CI 1.081–1.142) (Supplementary Table S2). The corresponding lower bounds were 1.34 (1.29) for PM_10_ and 1.06 (1.05) for green space. The relatively high E-values for PM_2.5_ and PM_10_ indicate moderate robustness to unmeasured confounding, whereas the low E-value for green space indicates greater sensitivity to residual confounding. Sensitivity analyses are reported in Supplementary Table S3. Although the P value trends for some pollutant-SUD and greenness-SUD associations showed fluctuations, the HR estimates were similar, and the associations did not change substantially after further adjustment.

**Fig. 2:**
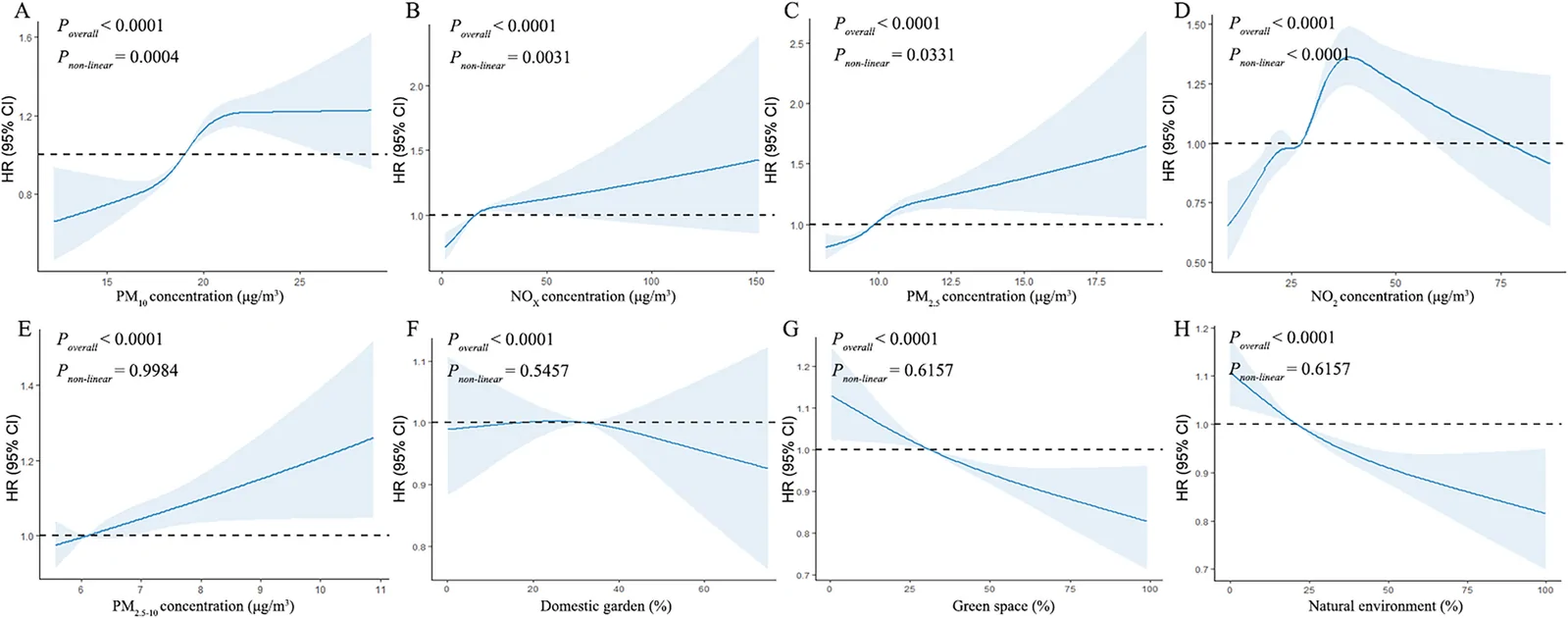
Smoothing curves of the relationship between air pollutants, green environments and substance use disorder. Dose-response association between PM_10_ (**A**), NO_X_ (**B**), PM_2.5_ (**C**), NO_2_ (**D**), PM_2.5−10_ (**E**), Domestic garden (**F**), Green space (**G**), Natural environment (**H**) and the risk of SUD evaluated by using restricted cubic spline regressions. Models were adjusted for age, sex, BMI, TDI, educational attainment, smoking status, alcohol consumption, and duration of residence

Restricted cubic spline models visualized the dose-response relationships (Fig. 2). For PM10 and NOX, the risk of substance use disorders rose steeply up to roughly 19 µg/m³ and then plateaued (*P*_*nonlinearity*_ = 0.0004 for PM_10_, 0.0031 for NO_X_). For PM_2.5_, risk increased steeply below approximately 9.6 µg/m³ and then increased more gradually (*P*_*nonlinearity*_ = 0.0331). PM2.5–10 showed no significant non-linear relationship (*P*_*nonlinearity*_ = 0.9984). For domestic garden, risk rose gently up to approximately 35% coverage and then declined gradually (*P*_*nonlinearity*_ = 0.5457).

### Effect modification by green environment on air pollutants-related SUD risk

**Fig. 3:**
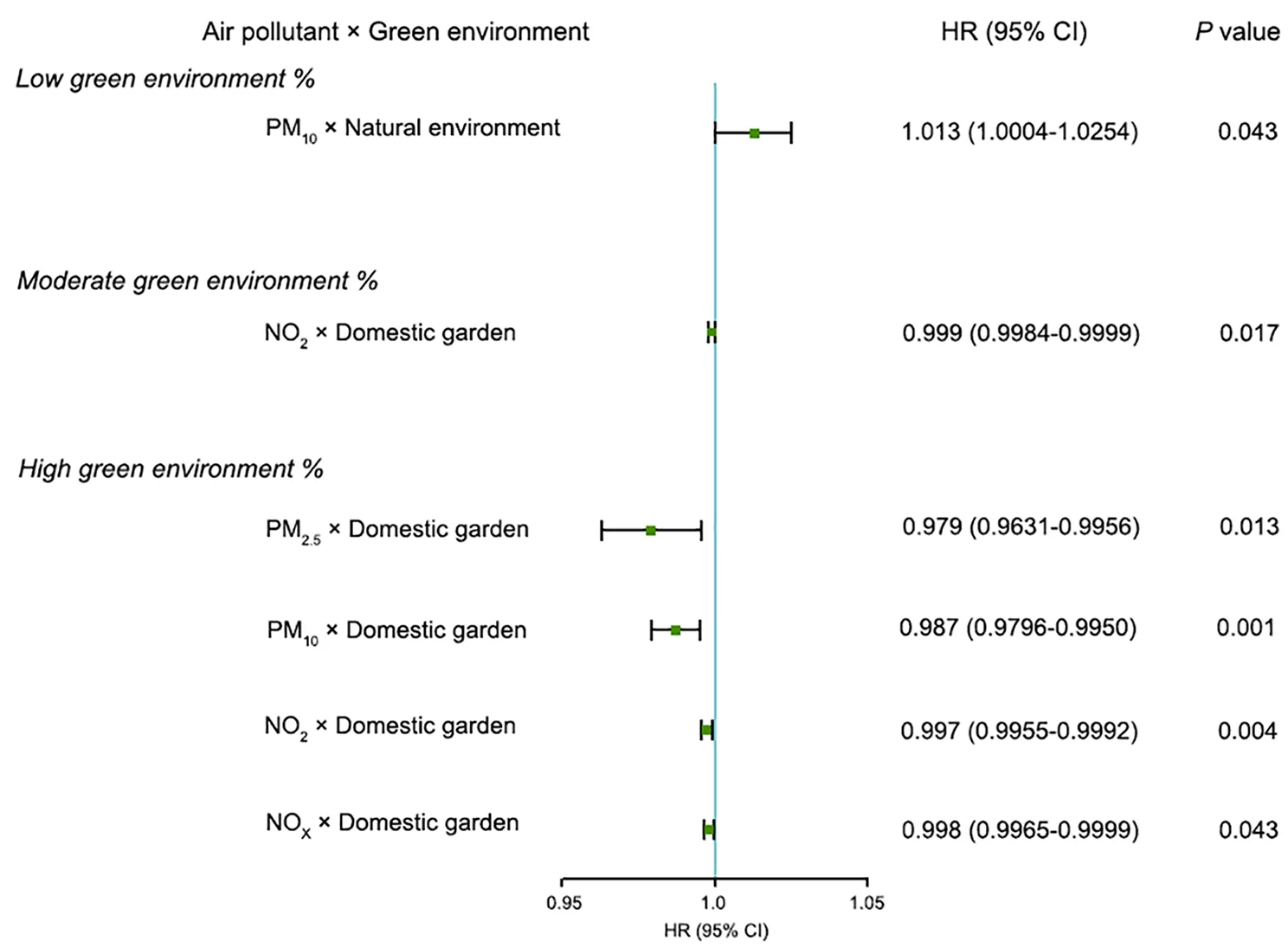
Associations of air pollutants and green environment on substance use disorder risk. Abbreviations: HR, hazard ratio; CI, confidence interval; PM_2.5_, particulate matter with an aerodynamic diameter ≤ 2.5 μm; PM_10_, particulate matter with an aerodynamic diameter of 10 μm; NO_2_: nitrogen dioxide; NO_X_, nitrogen oxides

Domestic garden showed consistent multiplicative interactions with several air pollutants in the high-exposure subgroup (Fig. 3). Cross-product HRs in the high domestic-garden group were 0.979 (95% CI 0.963–0.996, *P* = 1.29 × 10^− 2^) for PM_2.5_, 0.987 (95% CI 0.980–0.995, *P* = 1.36 × 10⁻³) for PM_10_, 0.997 (95% CI 0.996–0.999, *P* = 4.30 × 10^− 3^) for NO_2_, and 0.998 (95% CI 0.997-1.000, *P* = 4.28 × 10^− 2^) for NO_X_. Domestic garden also interacted with NO_X_ in the moderate-exposure subgroup (HR 0.999, 95% CI 0.998-1.000, *P* = 1.72 × 10^− 2^). Natural environment interacted with PM_10_ in the low-exposure subgroup (HR = 1.013, 95% CI 1.000-1.025, *P* = 4.35 × 10^− 2^). Green space did not show statistically significant interactions with any air pollutant across greenness subgroups (Supplementary Table S4).

### Effect modification by demographic and lifestyle factors

Stratified analyses indicated that males, participants aged ≥ 65 years, and individuals with a history of smoking showed stronger positive associations of air pollutants with SUD risk, and derived larger inverse associations of green space. Formal multiplicative interaction tests identified statistically significant interactions for sex, age, and smoking status (*P* for interaction < 0.05) (Supplementary Table S5-S10). In the sex-stratified analysis, all five air pollutants were more strongly associated with risk in males, whereas only PM_10_ showed a stronger association in females (Supplementary Table S5). Smoking-stratified analyses identified a significant NO_X_-SUD association in the ever-smoking subgroup (Supplementary Table S9). No significant multiplicative interactions were observed for BMI, alcohol intake, or illicit-substance use.

## Discussion

In this prospective cohort study, long-term exposure to ambient air pollutants was associated with a higher risk of incident SUD, while residential green space and natural environment were inversely associated with risk. Domestic garden attenuated the positive pollutant-SUD associations in the high-exposure subgroup, with PM_2.5_ and PM_10_ showing the strongest attenuation. The positive pollutant associations were strongest among males, participants aged ≥ 65 years, and individuals with a history of smoking.

Prior evidence on air pollution and substance use disorders is sparse and largely cross-sectional. Szyszkowicz and colleagues reported positive associations between several air pollutants and emergency-department visits for drug and alcohol abuse [26], and a UK Biobank cross-sectional study identified strong air-pollution associations with neurological multi-morbidity, including alcohol and substance dependence [27]. By contrast, an Italian cohort study found no clear association between long-term air-pollution exposure and incident mental disorders [28]. The present prospective design, larger sample, and composite outcome definition may explain the divergence. Mechanistically, controlled-exposure studies in rodents have shown that ambient particulate matter can alter reward preferences [29], a behavior linked to addiction vulnerability [30], and broader neuroscience literature implicates inflammation, neurotoxicity, and hormonal disturbance in the air-pollution–mental-health pathway [31, 32].

The inverse greenness associations are consistent with the limited prior literature. Residential greenness in childhood has been linked to lower risk of adult psychiatric disorders, with substance use disorders among the strongest associations [33], and a recent systematic review synthesized evidence that nature-based interventions have potential for both prevention and treatment of SUD [34]. Several mechanisms may underlie these associations. First, green spaces support physical activity, which reduces craving and substance use [35]. Second, they promote social cohesion and interpersonal contact, both protective against initiation and relapse [36]. Third, they reduce ambient air pollution, noise, and heat stress, thereby buffering chronic physiological and psychological load [37]. These mechanisms are not mutually exclusive, and the present data cannot distinguish among them.

The cross-product interaction between domestic garden and air pollutants is consistent with the hypothesis that vegetation mitigates pollutant exposure and its downstream effects. Previous studies have shown that higher green coverage reduces air-pollution concentrations [21], possibly through oxidative-stress and inflammatory pathways [38]. Direct removal of pollutants by vegetation is limited, but green barriers can attenuate pollutant dispersion, and greener areas tend to show lower measured pollutant concentrations [39, 40]. Beyond physical buffering, proximity to green space supports psychological recovery [41], which itself reduces stress-related substance use.

Sex, age, and smoking emerged as the strongest effect modifiers. Males showed larger positive pollutant associations, in line with prior clinical and preclinical evidence that sex chromosomes and sex hormones shape addiction vulnerability beyond oestrogen-linked pathways alone [42]. Males also showed larger inverse associations with greenness, plausibly because unhealthy lifestyle behaviors cluster more strongly in men [43], and the restorative potential of green environments therefore has more to gain. Smoking-stratified findings are consistent with the broader air-pollution literature showing that smokers carry higher pollutant-related risk [44]. The age-stratified finding for participants aged ≥ 65 years suggests cumulative exposure effects that warrant confirmation in cohorts with longer follow-up.

The dose-response patterns further refine interpretation. PM_10_ and NO_X_ showed plateau effects beyond roughly 19 µg/m^3^, suggesting biological saturation or compensatory mechanisms at higher exposures [45, 46], and implying that incremental reductions in moderately polluted settings may yield the largest absolute benefit. In contrast, PM_2.5_ showed sustained risk elevation without plateauing, with particularly steep increases below 9.6 µg/m^3^, consistent with reports that ultrafine particles trigger neuroinflammation via oxidative stress even at concentrations below 5 µg/m^3^ [47]. The divergence suggests that particle oxidative potential and chemical composition, not mass alone, drive neurobehavioural toxicity [48], and that regulatory frameworks should incorporate metrics beyond mass-based standards.

Our findings carry important implications for population-level prevention strategies. A systematic review by Bhatta et al. identified personal attitude, perceived risk, perceived behavioral control, and subjective norms as the strongest predictors of individual adoption of PM_2.5_ preventive measures, alongside the roles of negative emotions, social norms, and education [34]. These insights are particularly relevant to our study, as they suggest that public health interventions aimed at reducing SUD risk through air pollution mitigation could be enhanced by targeting these psychosocial determinants, for instance, through risk communication campaigns that emphasize the neurobehavioral consequences of air pollution, alongside structural interventions such as improving residential greenness.

Several limitations should be acknowledged. First, residual confounding by unmeasured occupational exposures, dietary patterns, physical activity, and genetic predisposition cannot be excluded, although consistency across sensitivity analyses supports the main estimates. Second, residential self-selection bias is plausible, because individuals who choose greener neighborhoods may differ in unmeasured health behaviors. Third, exposure was assessed using baseline annual averages from LUR models, which do not capture temporal variation or residential mobility, although sensitivity analyses restricted to long-term residents yielded consistent results. Fourth, approximately half of participants were excluded due to missing data, and the UK Biobank’s healthy-volunteer bias may limit generalizability. Fifth, our study does not include cases from the no formal diagnosis source. This limitation means our findings primarily reflect associations with severe or late-stage SUD requiring hospitalization, which may affect generalizability to milder or earlier-stage cases managed in primary care or community settings. Future studies incorporating primary care records and outpatient registries are warranted to capture the full spectrum of SUD severity and stage. The cross-setting consistency reported by a Nepalese nationwide study is encouraging [49], but the linear pattern observed there and the plateau effects observed here may reflect different baseline exposure levels and pollutant mixtures, and future work should test whether green-space protections generalize to lower-resource settings.

## Conclusion

In summary, our findings showed that long-term exposure to ambient air pollutants was associated with a higher risk of incident SUD. Residential green space and natural environment were inversely associated with risk, and domestic garden attenuated the positive pollutant associations in the high-exposure subgroup. These findings support integrating residential greenness into environmental frameworks for substance use disorder prevention, alongside continued efforts to reduce ambient air-pollution exposure.

## Supplementary Information

Below is the link to the electronic supplementary material.

Supplementary Material 1

Supplementary Material 2

Supplementary Material 3

Supplementary Material 4

Supplementary Material 5

Supplementary Material 6

Supplementary Material 7

Supplementary Material 8

Supplementary Material 9

Supplementary Material 10

## Data Availability

All data generated or analyzed during this study are included in this published article [and its Supplementary information files]. The Supplementary Information given below.

## Abbreviations

SUD: Substance use disorders
PM: Particulate matter
